# Randomised controlled trial of the short-term effects of OROS-methylphenidate on ADHD symptoms and behavioural outcomes in young male prisoners with attention-deficit/hyperactivity disorder (CIAO-II)

**DOI:** 10.1186/s13063-019-3705-9

**Published:** 2019-12-02

**Authors:** Philip Asherson, Lena Johansson, Rachel Holland, Tom Fahy, Andrew Forester, Sheila Howitt, Stephen Lawrie, John Strang, Susan Young, Sabine Landau, Lindsay Thomson

**Affiliations:** 1 Institute of Psychiatry, Psychology and Neuroscience, King’s College London, De Crespigny Park, London, SE5 8AF UK; 2 Healthcare Department, HM Prison Brixton, Jebb Avenue, London, SW2 5XF UK; 3 Division of Psychiatry, University of Edinburgh, Morningside Park, Edinburgh, EH10 5HF UK; 4 Psychology Services Limited, PO1735, Croydon, CR0 7WA UK

**Keywords:** Neurodevelopmental disorder, ADHD, OROS-methylphenidate, Prison mental health, Trial

## Abstract

**Background:**

Attention-deficit/hyperactivity disorder (ADHD) is a highly prevalent disorder, seen in 20–30% of young adult prisoners. Pharmacoepidemiological studies, a small randomised controlled trial and open trial data of methylphenidate suggest clinically significant reductions in ADHD symptoms, emotional dysregulation, disruptive behaviour and increased engagement with educational activities. Yet, routine treatment of ADHD in offenders is not yet established clinical practice. There is continued uncertainty about the clinical response to methylphenidate (MPH), a first-line treatment for ADHD, in offenders, who often present with an array of complex mental health problems that may be better explained by states of inattentive, overactive, restless and impulsive behaviours. To address this problem, we will conduct an efficacy trial to establish the short-term effects of osmotic-controlled release oral delivery system (OROS)-methylphenidate (Concerta XL), an extended release formulation of MPH, on ADHD symptoms, emotional dysregulation and behaviour.

**Methods:**

This study is a parallel-arm, randomised, placebo-controlled trial of OROS-MPH on ADHD symptoms, behaviour and functional outcomes in young male prisoners aged 16–25, meeting Diagnostic and Statistical Manual of Mental Disorders, fifth edition criteria for ADHD. Participants are randomised to 8 weeks of treatment with OROS-MPH or placebo, titrated over 5 weeks to balance ADHD symptom improvement against side effects. Two hundred participants will be recruited with a 1:1 ratio of drug to placebo. The primary outcome is change in level of ADHD symptoms after 8 weeks of trial medication.

**Discussion:**

Potential benefits include improvement in ADHD symptoms, emotional dysregulation, attitudes towards violence and critical incidents and increased engagement with educational and rehabilitation programmes. Demonstrating the efficacy and safety of MPH on ADHD symptoms and associated impairments may provide the data needed to develop effective healthcare pathways for a significant group of young offenders. Establishing efficacy of MPH in this population will provide the foundation needed to establish long-term effectiveness studies with the potential for demonstrating significant reductions in criminal behaviour and improved health-economic outcomes.

**Trial registration:**

ISRCTN registry, ISRCTN16827947, 31st May 2016; EudraCT number, 2015-004271-78, 31st May 2016. Last particpant last visit 6 June 2019. Data lock 27 August 2019.

## Background

Attention-deficit/hyperactivity disorder (ADHD) is characterised by developmentally inappropriate levels of inattentive, hyperactive and impulsive behaviours. The disorder is often accompanied by symptoms of emotional instability and leads to clinical and psychosocial impairments culminating in long-term negative outcomes and comorbid conditions [1]. ADHD affects around 5–7% of children [2, 3] and 3–4% of adults [4, 5]. Individuals meeting diagnostic criteria for ADHD are found at disproportionately high rates in prison populations with an estimated prevalence rate between 20 and 30% in young offender institutes and prisons [6].

Both the National Institute for Health and Care Excellence (NICE) and the Scottish Intercollegiate Guidelines Network (SIGN) recommend methylphenidate (MPH) for treating ADHD with significant impairment in children, adolescents and young adults [7–9]. Nevertheless, it is uncommon to diagnose and treat ADHD in young adult offenders. The reasons for this are unclear, but concerns have been expressed that the common occurrence of mental health, neurodevelopmental and psychosocial problems might provide a better explanation for impulsive, overactive and inattentive states in young offenders, or might interfere with the treatment response in cases of ADHD. For these reasons NICE [7] recommended that drug treatment efficacy trials are needed in offender populations (page 134 of the full guideline, section 5.18.1.4) and repeated this recommendation in 2013 [10]. The guideline stated that ’there should be an assessment of efficacy in these groups (i.e. forensic and drug abuse populations) of the ADHD treatments already recommended for treatment in the community. Randomised controlled trial design is recommended’. Clinical trials of ADHD treatments have yet to be conducted in young adult offenders, and the efficacy of MPH treatment for ADHD remains unknown in this group.

There are two main reasons why response of ADHD symptoms to stimulant medications may be different for young offenders compared to responses for previous studies in community ADHD samples. First, offenders present with an array of complex mental health problems that may better explain the states of inattentive, overactive, restless and impulsive behaviours used to define ADHD. These include problems commonly seen in offenders such as personality, anxiety, post-traumatic stress and substance abuse disorders, in addition to general and specific learning difficulties. Second, nearly all previous treatment trials of ADHD have been conducted in carefully selected samples with low levels of co-occurring psychosocial and mental health comorbidities. However, the co-occurrence of mental health disorders might modify the efficacy of drug treatments in ADHD. One example is comorbid drug abuse. Meta-analysis of treatment trials found no effect of MPH on ADHD symptoms in ADHD cases comorbid with drug abuse (standardised mean difference [SMD] = 0.08, *p* = 0.59), whereas there was a medium effect in non-comorbid samples (SMD = 0.51, *p* < 0.00001) [11]. This may be relevant to prison populations, where a history of drug abuse is common. Further, concerns have been expressed about the potential for stimulant medications such as MPH to worsen coexisting conditions. The most recent research recommendations from NICE [8] state that: ’no evidence was identified to justify different medication choices in people with ADHD and a history of psychosis, mania, or personality disorder. These groups are often excluded from trials. There are reasons (for example, mechanism of action of medication options, previous reports of adverse effects) to suspect that these groups may respond differently to different drugs, but a lack of trials to confirm this. Primarily there are some concerns that stimulant medication may worsen the symptoms of any of these coexisting conditions’.

### Choice of intervention and comparator

To address this problem, we are conducting a randomised controlled efficacy trial of osmotic-controlled release oral delivery system methylphenidate (OROS-MPH, Concerta XL), a sustained release formulation of methylphenidate, compared to placebo, in young adult prisoners with ADHD who are aged between 16 and 25 years.

### Previous studies

Previous community studies demonstrate the efficacy of MPH in reducing ADHD symptoms in children, adolescents and adults with ADHD [12]. A recent comprehensive network meta-analysis estimated an effect size from randomised controlled trials of MPH in reduction of ADHD symptoms in adults with an SMD of 0.78 (95% confidence interval [CI] of 0.62–0.93) [12]. However, there are only limited trial data for treatment of ADHD in young offenders presenting with a more complex mix of psychosocial, mental health and behavioural problems. One small randomised controlled trial of MPH in a prison sample of 30 Swedish prisoners with ADHD showed a large effect (SMD = 2.1) on ADHD symptom reduction [13]. While this study supports the treatment of ADHD in offenders, it cannot be considered definitive for the treatment of young offenders more generally, because of the small sample size, older age group and selection of offenders with severe ADHD and with long-term sentences treated in a special prison unit in Sweden.

Additional support comes from a pilot open label study that preceded this protocol; the study investigated the effects of MPH in 121 young offenders in HMP Isis in Southeast London who met Diagnostic and Statistical Manual of Mental Disorders, fifth edition (DSM-5) diagnostic criteria for ADHD [14]. That study followed similar procedures to the protocol reported here, but it was an open trial with a single treatment arm. Potential participants were screened using a DSM-IV symptoms checklist, and diagnosis was confirmed following the DSM-IV-based Diagnostic Interview for ADHD in Adults (DIVA) and medical review from a trained consultant psychiatrist. The prevalence of ADHD in the prison was estimated to be 19%, of which 78% met criteria for the combined type presentation of ADHD. Significant pre-post treatment reductions, unadjusted for multiple outcome measures, were seen for investigator-rated ADHD symptoms using the Conners Adult ADHD Rating Scale (CAARS).

More generally, the benefits of treating ADHD with MPH are also expected to extend to a wide range of long-term outcomes relevant to young adult offenders with ADHD. The evidence for this comes largely from pharmacoepidemiological studies using within-individual comparisons of periods on and off medication, to control for ‘treatment by indication’ effects. For example, suicidal behaviour was found to be higher among adults treated for ADHD with stimulants compared to those who had not been treated, suggesting that stimulants might increase the risk of suicidal behaviour [15]. However, reductions in suicidality were found when periods when individuals were taking medication for ADHD were compared to periods when the same individuals were not taking medication. This suggests a protective effective on suicidality during periods of taking stimulants, although individuals with ADHD and suicidality are more likely to be treated, presumably because their ADHD is more severe. Using a similar pharmacoepidemiological design, a study using Swedish national registry data of 25,656 male patients with ADHD found a sixfold higher rate of criminal convictions over a 4-year period in patients with ADHD compared to controls. Regarding medication effects, they found a 32% reduction in the risk of criminal convictions, using both within and between methods of analysis, to compare periods on and off medication for ADHD. Furthermore, these protective effects on criminal convictions were only seen for ADHD medications (stimulants or atomoxetine) but not for commonly prescribed antidepressants, indicating the specificity of these findings to ADHD medications [12]. Other outcomes identified using a similar methodology include violent reoffending on release from prison [16], depression [17] and risk of serious transport accidents [18].

In an earlier study of ADHD in prisoners, we found a sixfold increase in critical incidents among prison inmates with high levels of ADHD symptoms compared to prisoners with low levels of symptoms. This increase remained significant even after controlling for antisocial personality disorder [13], thus making this an important outcome for randomised controlled trials of prisoners with ADHD. Another important outcome is symptoms of emotional dysregulation such as irritability, anger and reactive aggression, which are also commonly seen in offenders with ADHD. Meta-analyses of randomised controlled trials of ADHD medications found reductions in emotional dysregulation, including problems with temper control, mood lability and emotional over-reactivity [19, 20]. Hence, treatment of offenders with ADHD might also lead to significant reductions in emotional dysregulation and potentially aggressive or violent behaviour. The symptoms of ADHD are also known to interfere with education and employment due to a combination of restlessness, reduced attention span, forgetfulness and problems with planning and organisation [21]. Treatment might therefore lead to greater positive engagement with prison educational and rehabilitation programmes. In our open label pilot study at HMP Isis, we also found significant effects on all the secondary outcomes proposed for this study (all *p* < 0.001) including measures of emotional dysregulation, attitudes towards violence, the number of critical incidents and positive engagement with the education and rehabilitation programme [14].

### Potential benefits

Potential benefits of treating young adult offenders with ADHD with MPH include improvement in clinical and behavioural outcomes. These include ADHD symptoms, emotional dysregulation, attitudes towards violence, critical incidents and engagement with educational and rehabilitation programmes. Demonstrating efficacy and safety of MPH on ADHD symptoms and associated impairments may provide the data needed to develop effective healthcare pathways, including the use of MPH, for a significant group of young offenders. Establishing efficacy of MPH in this population will provide the foundation needed to establish long-term effectiveness studies with the potential for demonstrating significant reductions in criminal behaviour and improved health-economic outcomes.

### Potential risks

One often-raised concern is the potential for abuse of prescribed MPH, particularly in a population of offenders with ADHD and high rates of substance abuse. Stimulants can be abused by crushing short-acting formulations such as immediate release MPH, which can then be insufflated (snorted) or injected, leading to a rapid entry of the drug into the brain and the experience of euphoria. However, when taken orally, the slow pharmacokinetic profile does not lead to euphoria [22]. This is important, because it is not possible to crush the trial medication (OROS-MPH, Concerta XL) or easily extract MPH for injection. Risk of diversion or abuse is therefore reduced in this study using this formulation of MPH. Furthermore, in our pilot study we did not observe excessive drug-seeking behaviour for stimulant medication [14]. The young adult offenders being treated for ADHD were generally cautious about increasing the dose of medication and were titrated to modest doses, comparable to community samples (18% used 18 mg daily, 37% used 36 mg, 14% used 54 mg, 26% used 72 mg and only 4% used 90 mg). There are standard operating procedures for the delivery of controlled drugs within the prisons. Other potential risks are the usual range of adverse effects observed when treating ADHD with OROS-MPH.

## Methods

### Trial design

**Fig. 1:**
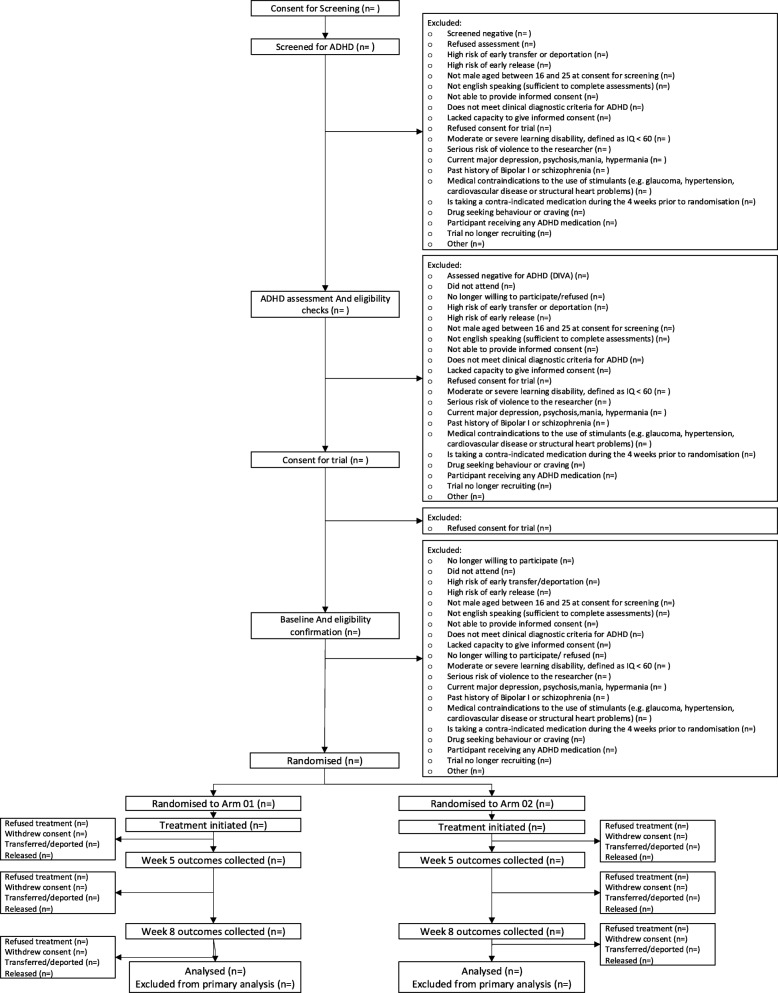
CONSORT diagram

The trial design is a parallel arm, randomised placebo-controlled trial of an extended release formulation of MPH (OROS-MPH, Concerta XL) on ADHD symptoms, behaviour and functional outcomes in young male prisoners aged 16–25 who meet DSM-5 criteria for ADHD. Participants will be randomised to 8 weeks of treatment with either OROS-MPH or placebo, titrated over 5 weeks to balance ADHD symptom improvement against side effects. Two hundred participants will be recruited with a 1:1 ratio of drug to placebo. The duration of each participant’s follow-up is 8 weeks from the start date of the trial medication. Figure 1 illustrates the prisoner’s journey through the trial as the Consolidated Standards of Reporting Trials (CONSORT) diagram. This process will be completed following database lock for the trial. Table 1 is a summary of all trial procedures and assessments. Additional file 2 provides the Standard Protocol Items: Recommendations for Interventional Trials (SPIRIT) checklist.

**Table 1:** Table caption

Visit	2	3	4	Randomisation and Initiation of treatment	5	6	7	8	9	10
Consent, randomisation and initiation of treatment										
Informed consent	X									
Eligibility checks			X							
Randomisation and initiation of treatment				X						
Treatment with OROS-MPH or placebo										
Treatment with OROS-MPH or placebo				X	X	X	X	X	X	X
Baseline (pre-randomisation), titration (visit 5-7), mediator (visit 9) and outcome (visit 10) measures										
DIVA (taken from pre-trial records)		X								
MINI 7.0.1 diagnostic interview		X								
MINI 7.0.1 cross disorder symptom ratings		X								X
Demographic data		X								
WASI-II (general cognitive ability)		X								
RPAQ		X								
CTQ		X								
Drug and alcohol use (AUDIT-C and NIDA)		X								
ZAN-BPD		X								
CAARS –O		X			X	X	X	X	X	X
AES			X		X	X	X	X	X	X
Blood pressure and pulse		X			X	X	X	X	X	X
WRAADS		X							X	X
ARI-S		X							X	X
MEWS		X							X	X
Demographic data		X								
MVQ		X								X
BSI		X								X
CGI		X								X
Weiss-CD		X								
Weight		X							X	X
Number of critical Incidents (adjudications)^b^		X								X
MOASP		X								X
MOASE		O								O
BRCP		X								X
BRCE		O								O
Educational engagement (proportion of education sessions attended)^b^		O								O
Positive and negative IEPs (HMP ISIS only)^b^		X								X
CORE-M		X								X
Concomitant medications and compliance^a^		X	X		X	X	X	X	X	X
Prescribed dose of trial medication and compliance^a^					X	X	X	X	X	X
Withdrawal status					X	X	X	X	X	X
Study medication guess										X

X indicates a measure which should always be recorded; O indicates a measure completed for a subset of participants participating in education and related activities; ^a^taken from prescription records; ^b^taken from

*Abbreviations*: *DIVA* Diagnostic interview for adult ADHD, *MINI-7.0.1* MINI international psychiatric interview for common mental health disorders, *WASI-II* Wechsler Abbreviated Scale of Intelligence-II, *RPAQ* Reactive proactive aggression questionnaire, *CTQ* Childhood trauma questionnaire, *AUDIT-C* Alcohol use screening test, *NIDA* NIDA quick drug screen, *ZAN-BPD* Zanarini borderline personality disorder scale, *CAARS-O* Observer rated DSM-IV ADHD, *AES* Adverse events scale, *WRAADS* Wender-Reimherr Adult ADHD Diagnostic Scale, *ARI-S* Affective reactivity index, *MEWS* Mind Excessively Wandering Scale, *MVQ* Maudsley violence questionnaire, *BSI* Brief symptom inventory, *CGI* Clinical global impression scale, *Weiss-CD* Conduct disorder scale, *MOASP* Modified overt aggression scale by prison officers, *MOASE* Modified overt aggression scale by education staff, *BRCP* Behaviour report cards by prison officers, *BRCE* Behaviour report cards by education staff, *IEPs* Incentive and Earned Privileges, *CORE-M* CORE outcome measure

### Trial objectives

The primary objective is to establish the efficacy of OROS-MPH in reducing ADHD symptoms (inattention and hyperactivity-impulsivity) in young male offenders aged 16–25 who meet diagnostic criteria for DSM-5 ADHD.

Secondary objectives include evaluating the following: reductions in emotional dysregulation, the number of adjudications for antisocial behaviour and rule breaking in the previous 8 weeks, ratings of aggressive and/or disruptive behaviour by prison officers and education staff, attitudes towards violence, self-report of well-being.

Additionally, we intend to investigate the hypothesis that improvements in secondary behavioural outcomes are mediated by improvements in ADHD symptoms or emotional dysregulation.

### Study setting

Participants are recruited from two prisons. The first is HMP & YOI Isis in London (England, UK), a prison for sentenced young adults and category C offenders, defined as those who cannot be trusted in open conditions but who are unlikely to try to escape. The second is HMYOI Polmont in Falkirk (Scotland, UK), a holding facility for young offenders aged 16–21, with sentences ranging from 6 months to life. All participants were sentenced prisoners when screened for entry into the trial.

### Eligibility criteria

#### Inclusion criteria

For inclusion into the study, one must be male, aged between 16 and 25 years (at consent for screening), English speaking (defined as sufficient to complete study assessments), able to provide informed consent (understand the information sheet and make an informed decision taking into account the pros and cons of study participation) and meeting clinical diagnostic criteria for DSM-5 ADHD.

The diagnostic criteria are defined as five or more symptoms of ADHD in either the inattentive or hyperactive-impulsive symptom domains, and six or more symptoms of ADHD in either the inattentive or hyperactive-impulsive symptom domains before the age of 12 years. Where it is not possible to gain enough clinical information to score childhood symptoms of ADHD, the operational criteria applied have been adapted to include evidence of several ADHD symptoms with impairment starting before the age of 12 years, and five or more symptoms currently with moderate to severe impairment. In addition this includes persistent trait-like (non-episodic) course of symptoms, impairments in two or more clinical or psychosocial domains and two or more settings from symptoms of ADHD and onset of symptoms before the age of 12 years.

#### Exclusion criteria

A subject is excluded from the study if he meets any of the following criteria. He lacks capacity to give informed consent; he has a moderate or severe learning disability, defined as IQ < 60; he poses a serious risk of violence to the researcher; he has current major depression, psychosis, mania or hypomania; he has a past history of bipolar disorder or schizophrenia (we exclude those with a clear history of episodic mania/hypomania or psychosis unrelated to acute drug intoxication, but do not exclude on the basis of chronic emotional dysregulation, i.e. irritability, frustration, anger or emotional-mood instability). Subjects are also excluded if they have medical contraindications to the use of stimulants (e.g. glaucoma, hypertension, cardiovascular disease or a structural heart problem); have been taking contraindicated medications during the 4 weeks prior to randomisation; show drug-seeking behaviour or craving (defined as drug-seeking behaviour that is unusually severe and likely to affect the titration protocol due to unusual and excessive demands for drugs or where there is a current withdrawal syndrome from an addiction disorder with drug dependency); receive any ADHD medication between consent for screening and randomisation.

### Trial medication

OROS-MPH (Concerta XL) is supplied as 18-mg capsules and placebo to match. Capsules are over-encapsulated and packaged in bottles of 46. Each bottle is assigned a unique randomisation number; the randomisation system allocates the right bottle to each patient. Over-encapsulation has been successfully adopted in previous studies to generate matched placebo to OROS-MPH. Piramal Healthcare UK Ltd. supply the investigational medicinal product (IMP), placebo to match manufacture, clinical trials packaging, Qualified Person (QP) Certification and distribution for 200 patients. The Sponsor arranged the supply of Concerta 18-mg tablets from the Marketing Authorisation Holder, Janssen-Cilag Limited. Janssen-Cilag Limited provides the summary of product characteristics (SmPC), updated throughout the duration of the study.

The over-encapsulated active tablets are repacked in high-density polyethylene (HDPE) bottles and exceed the remaining shelf life of the study without the need for a stability programme, as Concerta 18 mg has a Marketing Authorisation for both HDPE and blister packaging. Placebo tablets are manufactured once. Trial medication over-encapsulation and packaging is undertaken in two campaigns in order to accommodate a trial duration of up to 3.5 years. Concerta 18-mg tablets typically have a maximum shelf life of 3 years from the date of manufacture; however, by the time the product is repacked for the clinical trial, the remaining shelf life is likely to be under 2.5 years.

Over-encapsulation uses ‘DBcaps’ capsules which are designed specifically for the blinding of clinical trial medications. We have to over-encapsulate Concerta and placebo capsules with lactose capsules, rather than make a matching placebo capsule, because Concerta capsules have printing on them and are of a distinct shape that would be difficult to manufacture and might infringe copyright. We sought advice on this from previous investigators using OROS-MPH and from companies that provide drug and placebo supplies for studies. Studies on the use of DBcaps have shown that over-encapsulation of capsules results in a lag time of 2–3 min in disintegration compared with the unencapsulated capsules. The pharmacokinetic properties of Concerta XL 18-mg prolonged release capsules indicate release over several hours: following oral administration of Concerta XL to adults the drug overcoat dissolves, providing an initial maximum drug concentration at about 1–2 h. The MPH contained in the two internal drug layers is gradually released over the next several hours. Peak plasma concentrations are achieved at about 6–8 h, after which plasma levels of MPH gradually decrease (Section 5.2 of the Summary of Product Charateritics: https://www.medicines.org.uk/emc/product/6872/smpv).

### Prescribing and titration procedures

Trial medication is delivered as prescribed daily, with participants observed to ensure they swallow the capsules. There is a daily record of compliance with the trial prescription. Both active medication and placebo are titrated in the same way. Treatment starts at an initial dose of 18 mg (1 tablet) for 1 week and is then increased weekly over the following 4 weeks, in increments of 18 mg, to a maximum of 72 mg (4 tablets). Medication is reduced by 18 mg (1 tablet) if there is a limiting adverse event, in which case there will be no further increase in medication for the duration of the trial. Medication may be provided either once or twice daily up to the maximum daily dose. Titration upwards will be stopped if all 18 ADHD symptoms are scored as negligible (score of 0 or 1 on the CAARS) or absent. Unacceptable levels of adverse effects on the lowest dose of 18 mg might lead to a cessation of treatment in a few cases.

A maximum dose of 72 mg was included for this trial because previous clinical trials indicated that a proportion of adults respond better at higher doses without unacceptable levels of adverse events, and because current licensing for Concerta XL up to 54 mg is based on dose levels for children and adolescents, rather than adults. NICE recommends a daily dose of MPH in adults to a maximum of 100 mg per day [7], and for Concerta XL the British National Formulary (No 62, September 2011) recommends doses up to a maximum of 108 mg in adults.

### Strategies to improve adherence

We envisaged that adherence with allocated medication will present a challenge for around 20% of participants. Some offenders may not feel motivated to take the trial medication if they experience adverse effects or do not feel they are improving. They may also take medication intermittently because of the strict prison regime that allows for only a short time window for leaving their cells to obtain medication from the medicine hatch on the prison wings. The cases involving these persons are not expected to contribute to missing data. In our pilot study we accrued considerable experience in managing the expectations of offenders and providing the support needed to help participants adhere to the trial protocol. The following steps will be adopted to maximise adherence to medication:

1. In the pilot, minor adverse effects (13%) were the most common reason for non-adherence to medication. This was linked to the observation that this population may be more sensitive to minor adverse effects, particularly changes in appetite, than community populations, perhaps reflecting the importance of meal times to prisoners. To maximise adherence to the protocol and minimise this as a potential source of missing data, we will take care to identify the early signs of minor adverse effects such as appetite loss and adjust the medication dose accordingly.
2. Seven percent of the pilot sample did not wish to take medication in the mornings (08:00), which was the initial protocol followed in the pilot study. We then adjusted the protocol to allow for 12:00 medication for those who got up later in the day, worked mainly in the afternoons or had a strong preference for a 12:00 dosing, which resolved the problem. This flexibility in dosing time more accurately reflects dosing decisions in the community and provides a better match to patients’ daily routines.
3. During the pilot study, prison staff did not always let patients out of their cells to receive medication or remind participants to get up on time. To resolve this problem, we initiated the use of research staff whenever possible to assist in the delivery of medication by checking that prisoners were always out of their cells on time to receive trial medication.
4. In the pilot study, treatment was disrupted for the Ramadan festival for several participants. We will take care to check that participants are not started on trial medication where religious customs might interfere with adherence to the trial protocol.
5. In the pilot study, daily adherence to the trial medication reduced when participants were not reviewed weekly. One of the findings in the pilot study was the importance that prisoners gave to the weekly follow-up meetings when they can discuss their ADHD and response to the treatment process, in addition to completing study assessments. We will therefore offer weekly meetings with offenders throughout the 8-week trial.
6. Nurse support in addition to a research assistant and medical staff will ensure that offenders are given the support they need to adhere to the protocol.

### Concomitant treatments

Concomitant treatments are allowed with medications that are not contraindicated with MPH. All concomitant medications are recorded in the study database. Use of the following medications in the 4 weeks prior to the start of treatment with Concerta XL will lead to exclusion from the clinical trial, based on potential adverse drug interactions: clonidine, coumarins, monoamine oxidase inhibitors, moclobemide and rasagline.

### Baseline and outcome measures

The schedule of baseline and outcome measures as well as the procedures for the trial are listed in Table 1. Baseline-only measures are collected on all participants prior to randomisation and include descriptors of the study population and baseline moderators for further analysis as predictors of the treatment response. Primary and secondary outcome measures are collected at baseline prior to randomisation and 8 weeks after the initial trial prescription. The primary outcome is the investigator-rated Conners Adult ADHD Rating Scale (CAARS-O) at the 8 weeks outcome. The other 5-week and 8-week measures are secondary outcomes or mediator variables. All outcome measures are listed in Table 2 along with their definitions.

**Table 2:** Primary and secondary outcome measures

Concept	Instrument	Time points for analysis model	Type of measure	Summary measure
ADHD symptoms	Conners Adult ADHD rating scale (CAARS), investigator rated	M (B, 4, 5, 8)	Continuous scale	Mean difference
Emotional dysregulation	Wender-Reimherr Adult ADHD Diagnostic Scale (WRAADS), investigator rated	M (B, 5, 8)	Continuous scale	Mean difference
Irritability	Affective Reactivity Index (ARI), self-rated	M (B, 5, 8)	Continuous scale	Mean difference
Spontaneous mind wandering	Mind Excessively Wandering Scale (MEWS), self-rated	M (B, 5, 8)	Continuous scale	Mean difference
Attitudes towards violence	Maudsley Violence Questionnaire (MVQ), self-rated	M (B, 5, 8)	Continuous scale	Mean difference
Common psychopathological symptoms	Brief Symptom Inventory (BSI), self-rated	M (B, 5, 8)	Continuous scale	Mean difference
Global impression of disease severity	Clinical Global Impression (CGI) scale, clinician rated	M (B, 5, 8)	Categorical	Mean difference
Behavioural problems recorded by prison officers in prison records	Number of critical incidents recorded in prison records	Incident rate (B, 8)	Incident rate over 8-week period	Incidence rate ratio
Prison office ratings of aggressive behaviour	Modified Overt Aggression Scale (MOASP), Prison Officer report	M (B, 8)	Continuous	Mean difference
Educational staff ratings of aggressive behaviour	Modified Overt Aggression Scale (MOASE), Education Staff report	M (B, 8)^a^	Continuous	Mean difference
Prison officer ratings of behaviour	Behaviour Report Card (BRCP), Prison Officer report	M (B, 8)	Continuous	Mean difference
Educational ratings of behaviour	Behaviour Report Card (BRCE), Educational Staff report	M (B, 8)^a^	Continuous	Mean difference
Engagement with the educational programme	Number of educational sessions attended over 8-week period	Incident rate (B, 8)^a^	Incident rate over 8-week period	Incidence rate ratio
Incentive points for rewarding behaviour	Number of Incentives and Earned Privileges (IEPs)	Incident rate (B, 8)	Incident rate over 8-week period	Incidence rate ratio
Current psychological distress	CORE Outcome Measure (CORE-OM), self-rated	M (8)	Continuous	Mean difference

Notes on time points for analysis:

M refers to modelling mean 8-week outcomes

Numbers in brackets refer to the assessment time points of measures included in the model (B = baseline, 4 = week-4 data, 5 = week-5 data, 8 = week-8 data)

For incident rate variables (e.g. number of behavioural problem reports) the baseline data are from the 8 weeks prior to randomisation; for the 8-week outcome the data are from the 8-week period from the start of medication

^a^Indicates a measure completed for the subset of participants participating in education and related activities

### Investigator-rated measuresThe following investigator-rated meaures are used in the study:

- DIVA v2.0: DSM-IV-based Diagnostic Interview for ADHD in Adults [23]. DIVA 2.0. is a semi-structured interview assessment used to capture the diagnostic symptoms and criteria for DSM-IV ADHD. The diagnostic algorithm applied to these data was modified for DSM-5 criteria.
- MINI 7.0.1: Mini International Neuropsychiatric Interview for comorbid mental health disorders [24]. MINI 7.0.1 is a semi-structured interview assessment used to capture DSM-IV diagnostic criteria for common mental health disorders. Sections completed included the following: major depressive episode, suicidality, manic episode, hypomanic episode, panic disorder, agoraphobia, social anxiety disorder, obsessive-compulsive disorder, post-traumatic stress disorder, psychotic disorder and mood disorder with psychotic features, generalised anxiety disorder, antisocial personality disorder. In addition to diagnostic categories evaluated at baseline only, we collected cross-disorder symptom checklist scores at baseline and at the 8 weeks assessments.
- ZAN-BPD: Zanarini Rating Scale for Borderline Personality Disorder [25]. A validated scale for the assessment of borderline personality disorder, used as a baseline moderator variable.
- CAARS-O: Conners Adult ADHD Rating Scale for ADHD symptoms [26]. The 8 weeks CAARS-O assessment is the primary outcome measure for this study. CAARS-O was also used as a secondary outcome at week 5 and to assist the psychiatrist in titrating participants onto the optimal trial medication dose. CAARS-O consists of the 18 DSM-IV ADHD symptoms, rated on a 4-point Likert scale (0 not at all, never; 1 just a little, once in a while; 2 pretty often; 3 very much, frequently). This scale and other closely similar scales have been extensively validated as outcome measures in previous clinical trials of adult ADHD.
- WRAADS: Emotional dysregulation measured from the Wender-Reimherr Adult ADHD Diagnostic Scale [27]. We applied the emotional dysregulation items from an interview assessment of the WRAADS-ED, following previous publications on the treatment response of emotional symptoms in ADHD [20, 28].
- AES: Adverse Events Scale [29]. Scale of common adverse effects associated with stimulant medications for ADHD used with permission from the CADDRA website.
- CGI: Clinical Global Impression scale [30]. Scale used by the research psychiatrist to give an overall rating of clinical severity and a clinical impression of the clinical response and adverse effects of the trial medication.

### Participant self-rating scales

Self-rating scales are given to the participants for self-completion. The scale questions are usually read out to participants who give their response accordingly. The self-rating scales are:

- Reactive-Proactive Aggression Questionnaire (RPAQ) [31]. This scale is included as a baseline moderator capturing proactive and reactive forms of aggression.
- Weiss CD: Weiss conduct disorder scale. This scale is included to capture conduct disorder symptoms as a baseline moderator.
- Alcohol Use Disorders Identification Test (AUDIT-C) and National Institute on Drug Abuse (NIDA) quick screen. Alcohol and substance abuse checklist using the AUDIT-C and NIDA quick screen to capture drug and alcohol use in year prior to the current prison sentence. The NIDA quick screen was adapted from the single-question screen for drug use in primary care by Saitz and colleagues [32]. AUDIT-C is validated as a quick screen for alcohol use [33].
- Childhood Trauma Questionnaire (CTQ) [34]. Included as a potential moderator of the clinical response to MPH.
- Barkley ADHD (B-ADHD). Self-rating scale for DSM-IV ADHD symptoms [35] is as an initial screening instrument. Participants are considered to screen positive for ADHD if they have four or more symptoms scoring 2 or more in either the inattentive or hyperactive/impulsive symptom domain.
- Affective Reactivity Index (ARI-S) [36]. A self-rating scale for irritability.
- Mind Excessively Wandering Scale (MEWS) [37]. A self-rating scale that captures excessive spontaneous mind wandering, an aspect of psychopathology that is closely associated with ADHD and a strong predictor of ADHD-associated impairment in daily life.
- Brief Symptom Inventory (BSI) [38]. A self-rating scale that captures comorbid symptoms. Subscales include nine symptom dimensions: somatisation, obsession‐compulsion, interpersonal sensitivity, depression, anxiety, hostility, phobic anxiety, paranoid ideation, psychoticism.
- Maudsley Violence Questionnaire (MVQ) [39]. This scale was designed to capture beliefs associated with violence. The Machismo subscale relates to embarrassment over backing down and justification of violence in response to threat and attack; the Acceptance subscale includes the overt enjoyment and acceptance of violence in everyday life. In previous research the Machismo subscale showed the greater relationship to actual violence [39] and the greater reduction in our pilot study for this trial [14].
- CORE Outcome Measure (CORE-OM) [40]. This scale captures subjective well-being, problems and symptoms, life functioning and risk and harm. It is designed to measure psychological distress before and after treatment.

### Data from prison records and prison staff

Data will be collected from prison records and prison nursing and educational staff relating to behaviour in the 8 weeks before the collection of the baseline measures. For cases of individuals new to custody presenting with significant behavioural problems, the retrospective baseline reporting period will be for a period of 1 month or more, to allow for initial behavioural problems that may arise when people first enter prison. The data will include:

- Number of adjudications for antisocial behaviour and rule breaking (HMP Isis and HMP/YOI Polmont) and negative Incentives and Earned Privileges (IEPs) (HMP Isis only)
- MOASP: Ratings of aggressive behaviour by Prison Officers using the Modified Overt Aggression Scale (MOAS) [41]
- BRCP: Ratings of behaviour by prison staff using Behaviour Report Cards [42] by Prison Officers
- MOASE: Ratings of aggressive behaviour by Education Staff using the MOAS. This item is optional, depending on whether prisoners are attending education sessions or not
- BRCE: Classroom Behaviour Report Card scored by Education Staff (HMP Isis and HMP/YOI Isis). This item is optional, depending on whether prisoners are attending education sessions or not
- IEPs: Number of positive IEPs for positive engagement in education, occupational and rehabilitation programmes (HMP Isis only).

#### Baseline measures

The following measures are recorded at baseline. CAARS-O; WRAADS (three subscores: temper, affective lability, emotional over-reactivity), weight, pulse, blood pressure, Weiss CD, IQ (Wechsler Abbreviated Scale of Intelligence [WASI]), DIVA score and ADHD diagnosis, ZAN-BPD score, drug use in lifetime and alcohol use in the past year, RPAQ (two subscales: reactive, proactive), MEWS, CTQ, MVQ, CORE-OM, CGI, ARI-S, concomitant medications, BSI, MINI 7.0.1 and AES.

#### Primary outcome measure

The primary endpoint is the level of ADHD symptoms measured on the investigator-rated CAARS-O at 8 weeks post-treatment initiation to address the question of efficacy of OROS-MPH on ADHD symptoms in young offenders meeting DSM-5 diagnostic criteria for ADHD. Investigator-rated CAARS-O scores are a common outcome measure used in previous treatment trials of ADHD in the community; the CAARS-O score measures the same list of 18 symptoms used as the primary outcome in nearly all other studies of adult ADHD.

#### Secondary outcome measures

Secondary outcomes address important questions about the effects on comorbid symptoms and behavioural impairments that are commonly seen in offenders with ADHD. These are critical incidents (adjudications) from prison records for the 8-week period (in two 4-week periods) from initiation of the trial medication to the 8 weeks assessments; ratings of aggressive behaviour by prison staff using the MOASP at 8 weeks; BRCP behaviour report cards from prison staff at 8 weeks; engagement with educational activities (including number of scheduled educational sessions, proportion of scheduled educational sessions attended and reports of disruptive behaviour in education session reported at 8 weeks using the BRCE and MOASE completed by education staff—only for those people involved in education); attitudes towards violence using the MVQ at 8 weeks, CORE-OM at 8 weeks; general psychopathology using the BSI at 8 weeks; excessive mind wandering measured using the MEWS at 8 weeks; symptoms of emotional dysregulation measured using the WRAADS at 8 weeks; symptoms of emotional dysregulation measured using ARI at 8 weeks; overall health measured using CGI at 8 weeks.

#### Mediator measures

To address the secondary mediation hypotheses, the following putative mediators are recorded at 5 weeks and at baseline: CAARS-O hyperactive/impulsivity and inattention subscores and WRAADS for emotional dysregulation. These measures are hypothesised to mediate treatment response in terms of secondary behavioural outcomes (critical incidents and prison staff classroom report cards). Critical incidents are taken from the prison records at 8 weeks and are recorded over the previous 4 weeks. The prison staff classroom report cards are recorded at 8 weeks and record behaviour over the preceding week.

### Participant timelines

**Fig. 2:**
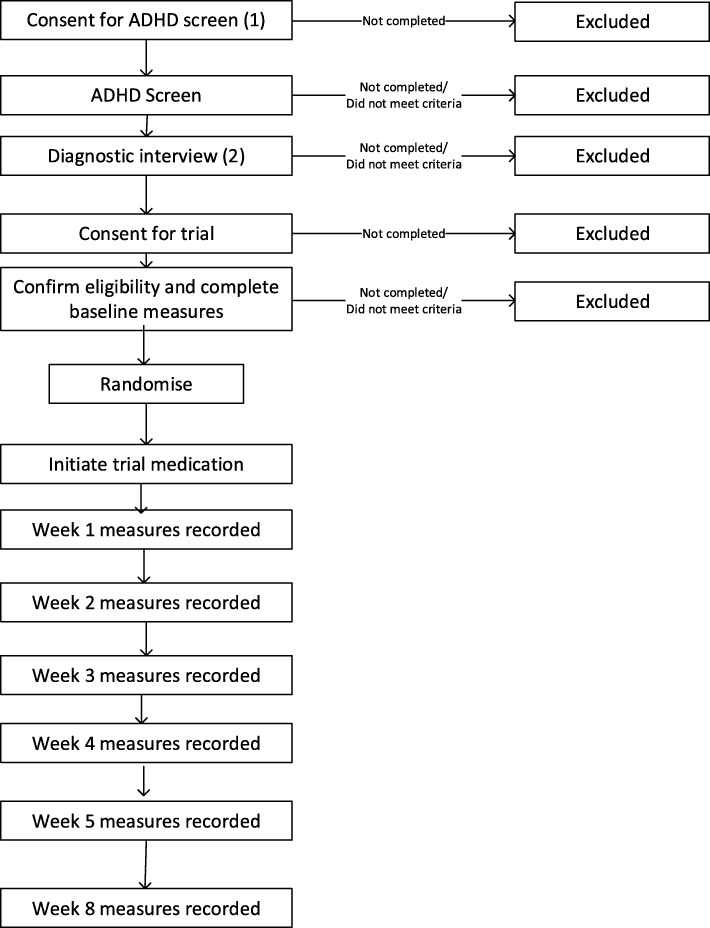
Schematic diagram of flow of participants (potential and actual) through the pre-trial assessment and trial

A schedule of participant visits is illustrated in Fig. 2.

#### Consent

There are two stages of consent. Initial consent 1 (screening and diagnostic step) allows for the use of screening questionnaires for ADHD, followed by a diagnostic assessment using the DIVA interview for adult ADHD and review by a trained psychiatrist. During these pre-trial steps, patients who fail eligibility criteria will not be invited to continue and will not be asked to provide consent 2, to participate further in the trial (clinical trial step). The eligibility criterion which they are identified as failing will be noted. Individuals who do meet the diagnostic and eligibility criteria are invited to take part in the clinical trial, at which stage informed consent is requested to take part in the randomised controlled trial. Consent 2 is taken by the trial psychiatrists.

Informed consent for the trial (consent 2) is obtained by providing the participants with written information sheets and ensuring that they understand the information provided and the implications of taking part in the research, prior to obtaining signed consent (see Additional file 1 for a copy of the information and consent forms). They are offered the opportunity to be treated for ADHD as part of the trial, or alternatively they are offered treatment outside of the trial. The participants have as long as they like to decide while the trial is ongoing.

Patients complete baseline measures after providing informed consent to take part in the trial (consent 2). Once the baseline assessments are complete and eligibility checks completed and documented, the individuals will be randomised to one of the treatment arms.

#### Research visits

Following consent 1 for screening, and confirmation of the diagnosis of ADHD and eligibility by a psychiatrist trained in the assessment of ADHD, information sheets and consent forms for the controlled trial (consent 2) will be provided and discussed with potential participants (visit 1). Information sheets will be reviewed and informed consent obtained for the clinical trial (visit 2). There is no limit on the time taken between visits 1 and 2 within the timeframe of the project. Potential participants will be encouraged to take as much time as they need to reach a fully informed decision about participation in the trial. Baseline data will be collected from participants, prison records and members of staff (visit 3). Once baseline data have been collected and eligibility confirmed following medical review by a psychiatrist, participants will be randomised to treatment with placebo or OROS-MPH (visit 4). Trial prescriptions will be completed and given to the pharmacy. Medication should start within 1 week of visit 4 (i.e. randomisation).

One week after the start of trial medication, the participants are reviewed, and trial medication titrated according to their clinical response and adverse effect profile (visit 5, week-1 titration). Symptoms of ADHD are measured using the CAARS-O, adverse events checked using the AES and blood pressure and pulse checked. This titration procedure is repeated at weeks 2–4 (visits 6–8). Five weeks after the start of medication (visit 9, week-5 assessment) the prescription (the titrated dose) is confirmed and maintained for the rest of the trial. At the week-5 assessment, outcome measures are completed by a research investigator for the CAARS-O, WRAADS and MEWS and the pulse, blood pressure, weight and AES. The final visit 10 is completed 3 weeks later after 8 weeks from the first prescription of the trial medication. At this visit all outcome measures are completed. As far as possible the information on clinical response derived during the titration visits (weeks 1–4) is not shared with other members of the research team, particularly with the investigator completing the weeks 5 and 8 outcome measures. Thus, potential unblindings based on the observed clinical response and adverse events will be minimised.

### Sample size calculations

The total sample size to be randomised is 200.

The primary outcome is ADHD symptoms, measured using CAARS-O. The results of a single-arm open label pilot study of young prisoners with ADHD who were given MPH showed a mean decrease of 25.0 points with a standard deviation of 9.1 [14]. This suggested a standardised effect size of SMD = 2.75. It could reasonably be assumed that at least 20% of this effect might be attributed to the effects of MPH. On this basis, this study is powered to detect a standardised effect size of *d* = 0.55. Assuming a standard deviation of 9.1, this would translate into a treatment difference of 5.0 points. This effect size is consistent with the results of a recent meta-regression analysis (12), which estimated the effect of treatment to be SMD = 0.49 (95% CI 0.08, 0.64). The sample size calculation used G*Power version 3 and was based on the use of a *t* test to compare the means of the treatment groups. In order to have 90% power at the 5% significance level to detect a standardised effect of SMD = 0.55, this study would need to collect outcome data on 142 participants. Inflating for the expectation that loss to follow-up may be as high as 25%, a minimum of 190 participants should be recruited, with the target for the study set at 200.

A 25% loss is expected to be easily achievable, since in the pilot 10% left the prison due to unexpected transfers from the prison, and problems with adherence to trial medication were rarely followed by problems completing trial assessments.

### Recruitment procedures

Participants will be recruited from HMYOI Isis (London) and HMYOI Polmont (Falkirk). Following consent to be screened for ADHD (consent 1), screening questionnaire data will be collected by the prison mental health teams using a DSM-IV ADHD symptom rating scale (25). Patients who screen positive will be invited to complete the DIVA [27]. This will be followed by a clinical review by a psychiatrist trained in the diagnostic assessment of ADHD, including collateral information obtained from an informant whenever feasible. Following clinical review, patients who meet diagnostic criteria for ADHD and the other eligibility criteria for the trial will be invited to take part in the clinical trial.

Eligibility for the study will be further checked and recorded once the consent form (consent 2) has been signed and baseline assessments have been completed, prior to randomisation. Using an algorithm that applies the DSM-5 criteria to the DIVA interview data, the potential participants will be checked to ensure they meet diagnostic criteria for DSM-5 ADHD. A clinical review by a psychiatrist trained in the diagnostic assessment of ADHD will review all inclusion and exclusion criteria. The exclusion criteria of IQ less than 60 will be based on the 95% CI for the IQ estimate from the WASI-II including IQ of 60, in combination with a clinical assessment by the psychiatrist to confirm that the participant has the ability to understand the rating scale and interview assessment questions; understand the information sheet and the study procedures and risks; and the ability to provide sufficiently detailed accounts of ADHD symptoms and behaviours, consistent with an IQ score greater than 60. Since there are no validated IQ tests for the visually impaired, including WASI-II, this criterion will be based on clinical judgement alone for participants with this impairment. This will also be the procedure for anyone unable to complete the WASI-II assessment due to severity of their ADHD symptoms or other mental health problems.

### Withdrawal of subjects

Participants have the right to withdraw from the study at any time for any reason, and healthcare staff have the right to withdraw patients from the trial if they consider the trial is having an adverse effect on the participants. However, where participants discontinue taking trial medication, we will invite them to remain in the study to complete trial assessments, thereby minimising loss of data. Should a participant decide to withdraw from the study, all efforts will be made to report the reason for withdrawal as thoroughly as possible.

Due to potential concerns about the interaction of trial medication with unknown psychoactive substances, if a participant discloses to any member of the research team that he has used ’spice’, i.e. synthetic cannabis or another unknown psychoactive substance, while participating in the study, a clinical evaluation will be made. If it is current use (defined as within the last 2 days), the study medication will be stopped. If it happened earlier in the study and is considered an isolated incident, the trial medication can continue. If the trial medication is stopped, the participant will remain in the study and will be asked to complete trial assessments. A clinical assessment will be made on a case-by-case basis as to the safety of restarting the trial medication after 48 h from the time of stopping the trial medication.

### Randomisation and allocation concealment

Randomisation to OROS-MPH or placebo will be at a 1:1 ratio. Randomisation is at the participant level and is performed using the King’s Clinical Trials Unit (CTU) independent Randomisation Service, ensuring reliability and credibility in the randomisation process, with blinding of both investigators and participants. Randomisation is stratified by prison with variable block sizes to ensure that equal numbers of patients are allocated to the two arms within each prison stratum. Patient characteristics will not be considered in the randomisation process. However, we expect the drug treatment and placebo trial arms to be balanced in terms of cognitive ability, ADHD symptom severity and co-occurring psychosocial and mental health problems.

Prescriptions are completed by the trial psychiatrist. Each patient is allocated a kit (labelled carton) containing four labelled bottles, each containing 46 active or placebo tablets. Each kit and its bottles will be labelled according to Annex 13 guidelines and have its own randomisation/treatment pack number. The centralised randomisation system will allocate the correct treatment pack/kit to each patient during the trial.

### Blinding

Blinding is maintained for all study investigators, including the on-site researchers, pharmacy, statistical and data management teams. Investigators will be unblinded after the primary analysis is complete. The primary analysis dataset will not include any trial medication dosage data to ensure that the statistician remains blinded. We do however propose a sensitivity analysis to assess efficacy for those complying with tablets offered. This analysis will exclude those participants who took no trial medication on less than 75% of the days on which it was prescribed. Additionally, persons who withdraw from treatment or the trial or are released, transferred or deported will be excluded. We will not consider what proportion of the prescribed medication was taken on any given day.

We intend to use linear mixed modelling, which assumes that only variables included in the model predict missingness. We will assess empirically whether this particular missing at random (MAR) assumption is reasonable, using an independent statistician to maintain blinding if necessary. If the assumption is not reasonable, multiple imputation will be used instead to accommodate the missing data generating process, and the statistician might need to become unblinded at this point, but investigators will remain blind until the primary analysis is complete. The Investigator must report all code breaks (with reason) as they occur on the case report form.

### Emergency unblinding

Emergency unblinding will follow the standard operating procedures for the King’s Health Partners Clinical Trials Office (KHPCTO). In circumstances where unblinding is deemed necessary, the starting point will always be the local investigating team. Whenever possible the decision to unblind will be made by the Chief Investigator, the Principal Investigator or clinically qualified staff working on the project. Out of hours, if clinically qualified members of the research team are not available, then the 24-h Emergency Scientific and Medical Services (ESMS) system will be used. The ESMS system consists of a call centre which is manned around the clock by information scientists who have a minimum qualification of a life science degree to include toxicology or pharmacology. These information scientists are always available and are the direct line of communication to the number on the patient card. The information scientists will be trained in the specific details of this study and have direct access to one of the ESMS consultant physicians should clinical advice be required. Our consultant physicians practice general and internal medicine and specialise in clinical pharmacology and toxicology, ensuring clinical advice is available night and day. To maintain the overall quality and legitimacy of the clinical trial, code breaks will occur only in exceptional circumstances when knowledge of the actual treatment is absolutely essential for further management of the patient. The Investigator will always maintain the blind as far as possible.

### Statistical analyses

A detailed statistical analysis plan has been developed by the trial statisticians in collaboration with the Chief Investigator and approved by the Trial Steering Committee (TSC). Analyses will be carried out by the trial statistician (RH) and checked by the senior statistician co-investigator (SL).

In the first instance data will be analysed under intention-to-treat assumptions; that is, participants will be analysed in the groups to which they were randomised irrespective of treatment received. Efficacy will be assessed by comparing primary and secondary outcomes between the OROS-MPH and placebo arms.

In order to assess the efficacy of the continuous primary CAARS-O outcome (see Table 2), a linear mixed model will be used. The model will contain CAARS-O scores from the last three (4, 5 and 8 weeks) post-randomisation time points as the dependent variable and baseline CAARS-O values, randomisation stratifier (prison), trial arm, dummy variables coding assessment time point (4, 5 or 8 weeks) and trial arm x time point interaction terms as explanatory variables. Random effects that vary at the participant level will be used to model the covariance structure between the repeated measures. The approach will ensure that a different trial arm effect can be estimated at 4, 5 or 8 weeks—with the estimated effect at 8 weeks providing the evaluation of OROS-MPH efficacy in terms of the primary outcome. Similar models will be used to evaluate continuous secondary outcomes.

The secondary count outcomes at 8 weeks (e.g. number of critical incidents) will be compared between treatment arms using Poisson regressions to estimate incidence rate ratios (after conditioning on baseline counts and randomisation stratifiers). Logistic regression will be carried out for scheduled educational sessions attended. Parameters will be estimated using maximum likelihood.

Inferences will remain valid in the presence of missing data provided that the missing data generating mechanism is MAR. More specifically, this particular MAR assumption stipulates that only variables included in the analysis model drive missingness. While we model several time points simultaneously, inferences will be made only at the time point of interest (8 weeks). Using linear mixed models means that we can allow variables measured and included in the model (e.g. previously observed values of the outcome including baseline values, trial arm, stratifier and post-treatment time point) to predict attrition, and allows us to make use of all available data. We will also check empirically whether withdrawal from allocated treatments is predictive of missingness at 8 weeks. And if we find that such post-randomisation variables drive missingness, we will consider using multiple imputation to accommodate such a MAR process. Mediation analysis using structural equation modelling will be used to partition the total treatment effect into mediated and non-mediated components.

## Discussion

### Accountability for trial medication

All aspects of treatment and accountability for managing the medication storage and delivery are managed locally by the prison pharmacies and mental health teams, as per standard practice for this medication in the prisons. Investigational medicinal product (IMP) accountability will be recorded and verified. All aspects of treatment compliance and recording of treatment administration/refusal are managed by the prison mental health teams and locally by healthcare staff as per standard practice for these sites. Patients are observed when they are given medication and checked to ensure the capsules have been swallowed. This information is then recorded (signed off) by nursing staff who delivered the medication on prison pharmacy record sheets or digital records.

### Safety checks

Patients are monitored daily by the prison mental and healthcare teams. Safety checks will be conducted in line with NICE Guidelines (2009).

Regarding the research aspect of the study (i.e. obtaining follow-up data), there is little risk to participant safety. Participants will be aware that should they wish to withdraw from the study they may do so. Participants who become upset or distressed by the questions in the research (this is unlikely, as the questions are similar to those asked regularly in the context of their clinical care) will be offered support by the researchers and by the prison mental health team.

The healthcare team will follow national guidelines on safety, which is predominantly related to monitoring of cardiovascular function. More specifically, the clinical care will follow these procedures:

1. Before commencing treatments, checks will be made on pulse and blood pressure and review of healthcare records.
2. Potential cardiovascular abnormalities will be evaluated for risk and, if necessary, an opinion will be obtained from a cardiologist prior to commencing treatment.
3. The clinical team will check pulse and blood pressure once a week for the first 5 weeks and at the end of the 8-week trial.

Other safety checks will include monitoring of adverse events during assessments. In addition, participants will be monitored daily by prison staff, and any potential adverse events will be reported to the prison healthcare team.

### Procedures for recording and reporting adverse events

Safety will remain the responsibility of the prison mental healthcare team. Adverse events of any medical or non-medical intervention identified or recorded by the research team at each site will be verified by the clinician who is part of the research team, or by an assigned medical colleague at specialist registrar grade or above who is a member of the prison healthcare team or by the clinical lead for the project (Professor Asherson). The decision to stop treatment following an adverse event will remain the responsibility of the clinical team. Minor adverse events that do not come under official reporting procedures will be reported to the clinical team, e.g. sleep disturbance, minor levels of anxiety or dysthymia, small increase in pulse and blood pressures, reduced appetite and other minor physical symptoms that do not endanger patients or cause more than minor distress. All other adverse events from medication will be recorded and reported in line with The Medicines for Human Use (Clinical Trials) Regulations 2004 and Amended Regulations 2006.

The research team acting on behalf of King’s College London as Sponsors have delegated the delivery of the Sponsor’s responsibility for Pharmacovigilance, as defined in Regulation 5 of the Medicines for Human Use (Clinical Trials) Regulations 2004, to the KHPCTO. Reporting of serious adverse events (SAEs) will continue until the last patient last dose has been completed. For each participant, the reporting period will be from the time of first dose of the trial medication to the end of his involvement in the trial (last dose at the end of 8 weeks). All SAEs, serious adverse reactions (SARs), suspected unexpected serious adverse reactions (SUSARs) and Important Medical Events (IMEs) (excepting those specified in this protocol as not requiring reporting) will be reported immediately by the Chief Investigator or designated site investigators to the KHPCTO in accordance with the current Pharmacovigilance Policy. We will copy this information to Janssen-Cilag at the same time.

The KHPCTO will report SUSARs and other SARs to the Medicines and Healthcare products Regulatory Agency (MHRA) and competent authorities of other European Economic Area (EEA) states in which the trial is taking place.

The Chief Investigator will report to the relevant ethics committees. Reporting timelines are as follows:

- SUSARs which are fatal or life-threatening must be reported not later than 7 days after the Sponsor is first aware of the reaction. Any additional relevant information must be reported within a further 8 days.
- SUSARs that are not fatal or life-threatening must be reported within 15 days of the Sponsor first becoming aware of the reaction.

The Chief Investigator and KHPCTO (on behalf of the Co-Sponsors) will submit a Development Safety Update Report (DSUR), relating to this trial IMP, to the MHRA and Ethics Committee annually.

### Treatment stopping rules

The trial may be prematurely discontinued by the Sponsor, Chief Investigator or Regulatory Authority on the basis of new safety information or for other reasons given by the Data Monitoring and Ethics Committee (DMEC)/TSC, Regulatory Authority or Ethics Committee concerned. Trial discontinuation for safety reasons is not envisaged given the successful pilot study. If the study is prematurely discontinued, active participants will be informed and no further participant data will be collected.

### Trial Steering Committee

A TSC will be convened to provide overall supervision of the trial and ensure the trial is conducted to the rigorous standards set out in the Medical Research Council (MRC) guidelines for Good Clinical Practice (GCP). The TSC will monitor progress, adherence and safety. The TSC chair is Professor Jenny Shaw (Consultant Forensic Psychiatrist, University of Manchester), and members include Dr. Ylva Ginsberg (Consultant Psychiatrist specialising in ADHD in prisoners, Stockholm, Sweden), Peter Mason (Forensic Psychiatrist and specialist in ADHD, Cheshire and Wirral), Anthony Davis, R&D Manager (Oxleas National Health Service [NHS] Foundation Trust), Dr. Ulrich Muller-Sedgwick (Barnet, Enfield and Haringey Mental Health NHS Trust) and Beverley Nolker, (POA learning and user representative). Non-independent members are the lead applicants in London and Edinburgh (Philip Asherson and Lindsay Thomson). Other members of the research management group will attend as observers and to report to the TSC. It is envisaged that the TSC will meet before the start of the project and every 6 months, alternating between telephone conference and face-to-face meetings.

### Data Monitoring and Ethics Committee (DMEC)

A DMEC will be convened to monitor the safety, efficacy, ethical conduct and quality of the data. The committee will consist of three members experienced in clinical trials, including an independent statistician. The DMEC chair is Professor Seena Fazel, University of Oxford (an experienced Forensic Psychiatrist). Other members are Professor Chris Hollis, University of Nottingham (an expert on the Clinical Management of ADHD) and Adrian Cook (a trial statistician). DMEC meetings will be timed to occur prior to TSC meetings for timely reporting to the TSC. A summary of the role and reporting structure is available from the Chief Investigator on request.

### Local trial management

The project will be led by Professor Asherson (PA) in London supported by a Trial Manager. The Trial Manager will liaise with the trial monitors and ethical board where required and support completion of interim reports as well as the ongoing management of the project. The Research Psychiatrist at YOI Isis will coordinate all daily activities on site. Principal Investigator Thomson (LT) will lead the project in Edinburgh and will be supported by the local RA, who will conduct similar day-to-day coordinating tasks in HMYOI Polmont.

### Project Management Group

The project will be led by Chief Investigator Asherson in London. The programme manager in London will liaise with the Edinburgh study coordinator weekly throughout the project, monitor progress and maintain communication about successes and barriers to progress and report back to the lead applicants. Asherson and Thomson will hold a weekly telephone conference to review progress with the data collection teams. A meeting of all investigators and co-applicants will review progress on a monthly basis.

### Ethical issues specific to this project

The trial will be conducted in compliance with the principles of the Declaration of Helsinki (1996), the principles of GCP and in accordance with all applicable regulatory requirements including but not limited to the Research Governance Framework and the Medicines for Human Use (Clinical Trial) Regulations 2004, as amended in 2006 and any subsequent amendments. This protocol and related documents will be submitted for review to London South East Research Ethics Committee (REC), and to the MHRA for Clinical Trial Authorisation. Annual progress and safety reports and a final report at conclusion of the trial will be submitted to the KHPCTO (on behalf of the Sponsor), the REC and the MHRA within the timelines defined in the regulations. We previously received ethical approval and MHRA registration for the current open label trial of OROS-MPH in HMP Isis and will follow the same recruitment and consent procedures as in the previous study.

OROS-MPH is only licensed for first-time use in young people with ADHD and severe impairment under the age of 18, although NICE also recommends MPH as the first-line treatment for ADHD in adults. The 8-week trial includes a placebo group, so we will be denying a recommended treatment for ADHD during this period. However, currently prisoners with ADHD are rarely treated because of uncertainty over validity of the ADHD diagnosis, efficacy of treatment and concerns about potential drug abuse and diversion in prison populations. To address the issue of equal access to treatment, we will offer treatment to all participants once the trial is completed. Care will be taken to ensure that no coercion is involved in recruiting prisoners into the study. Initial consent will be obtained by members of the prison mental health team. Following procedures in the pilot study, informed consent will be obtained at the screening and diagnostic steps as well as at the start of the trial. All participants will have the mental capacity to make informed decisions. It will be made very clear that taking part in the study will have no impact, negative or positive, on their time in the prison or the prison regime. However, some participants may benefit (and show improvements in behaviour) from the treatment that is offered as part of the clinical trial. Taking part in the study will not lead to loss of earnings. The study medication is a controlled substance. There are however standard operating procedures in place for the prescription of controlled drugs from the prison pharmacy.

Participants are informed that their anonymised research data from this study will be stored securely and may be shared with other scientists or research groups where this helps us to understand the findings of the study. The data may also be used in combination with data from other similar studies.

All personal information is stored in a secure place (locked cabinet in locked office in the prisons). Outside of the immediate healthcare team involved in this research, no one will be able to match personal information (name and prison number) with the information gathered for research. After completion of the study, personal information held by the local study teams will be destroyed. Personal details will only be used to contact participants about the study. Personal details will not be linked to clinical or prison records in the research records. The clinical data and prison records used for research will be identified using a study ID code.

### Quality assurance

Monitoring of this trial will be to ensure compliance with GCP, and scientific integrity will be managed and oversight retained by the KHPCTO Quality Team.

### Data handling

The Chief Investigator (PA) will act as custodian for the trial data. Data will be stored on a database to be set up by the Clinical Trials Unit (CTU) at King’s College London. Patient data will be pseudo-anonymised. All pseudo-anonymised data will be stored on a password-protected computer. All trial data will be stored, handled, processed and archived in line with the Data Protection Act and the Medicines for Human Use (Clinical Trials) Amended Regulations 2006.

### Data management

Data will be stored on a trials database to be set up by the CTU at King’s College London. This allows for full audit information and checks on data entry that will be used to ensure the integrity of the data collection and monitoring of the study progress. At the end of the study, all research records will be transferred by secure courier service to the Social, Genetic and Developmental Psychiatry (SGDP) Centre, King’s College London. A detailed data management plan is available from the authors.

### Publication policy and access to data

It is intended that the results of the study will be reported and disseminated at international conferences and in peer-reviewed scientific journals. Additionally, we will disseminate through internal reports to the prison services and through relevant online forums such as the UK Adult ADHD Network (UKAAN). The data collected will be shared and used in collaborative studies once the main objectives of the study have been completed. We will provide efficient and rapid access to data to scientists following a formal review process by the study co-investigators. The dataset that will be shared will be anonymised, meaning that it will not include data of name, birthdate or prisoner record number. Any raw data from the trials database or randomisation system will not be shared. Investigators who wish to access the data once the study is finished should contact the Chief Investigator (PA) and request permission. A formal application will be required indicating the question to be addressed and methods to be applied. Data access will be allowed for all legitimate scientific enquiries following scientific review by the Chief Investigator and co-applicants.

Over the duration of the study, many people will have contributed to the CIAO-II project. We will follow the principles outlined in the International Committee of Medical Journal Editors Recommendations for the Conduct, Reporting, Editing, and Publication of Scholarly Work in Medical Journals (ICMJE Recommendations 2013).

These four criteria are reproduced as follows:

1. Substantial contributions to the conception or design of the work; or the acquisition, analysis, or interpretation of data for the work; AND
2. Drafting the work or revising it critically for important intellectual content; AND
3. Final approval of the version to be published; AND
4. Agreement to be accountable for all aspects of the work in ensuring that questions related to the accuracy or integrity of any part of the work are appropriately investigated and resolved.

In addition to being accountable for the parts of the work he or she has done, an author should be able to identify which co-authors are responsible for other specific parts of the work. In addition, authors should have confidence in the integrity of the contributions of their co-authors.

### Insurance/indemnity

Clinical Trial insurance is provided by the King’s College London Clinical Trials Insurance Policy.

### Trial status

The protocol version is version v2.0, 30 August 2018. Recruitment started 30 May 2016. Recruitment was completed on 30 May 2019. Last particpant last visit 6 June 2019. Data lock 27 August 2019.

## Supplementary information

**Additional file 1.** : Information sheet and consent form for the screening stage and trial stage of the study. (DOCX 44 kb)

**Additional file 2.** : SPIRIT 2013 checklist: recommended items to address in a clinical trial protocol and related documents. (DOC 122 kb)

## Data Availability

It is intended that the results of the study will be reported and disseminated at international conferences and in peer-reviewed scientific journals. Additionally, we will disseminate through internal reports to the prison services and through relevant online forums such as UKAAN. The data collected will be shared and used in collaborative studies once the main objectives of the study have been completed. We will provide efficient and rapid access to data for secondary analyses. We will provide access to scientists following a formal review process by the study co-investigators, to minimise duplication of effort by different research teams. We will encourage teams with similar research questions to work collaboratively. The dataset that will be shared will be anonymised, meaning that it will not include the name, birthdate, prisoner record number or other data that could potentially identify individuals. In the initial phase of data sharing, investigators who wish to access the data once the study is finished should contact the Chief Investigator (PA) and request permission. A formal application will be required indicating the question to be addressed and methods to be applied. Data access will be allowed for all legitimate scientific enquiries following scientific review by the Cchief Investigator and co-applicants.

## Abbreviations

ADHD: Attention-deficit/hyperactivity disorder
AES: Adverse Events Scale
ARI-S: Affective Reactivity Index
AUDIT-C: Alcohol Use Disorders Identification Test
BRCE: Behaviour Report Card by Education Staff
BRCP: Behaviour Report Card by Prison Officers
BSI: Brief Symptom Inventory
CAARS: Conners Adult ADHD Scale
CAARS-O: Conners Adult ADHD Rating Scale
CGI: Clinical Global Impression
CORE-OM: CORE Outcome Measure
CTQ: Childhood Trauma Questionnaire
CTU: Clinical Trials Unit
DIVA: Diagnostic Interview for Adult ADHD
DMEC: Data monitoring and ethics committee
DSM: Diasgnostic and statistical manual
DSUR: Development Safety Update Report
ESMS: Emergency Scientific and Medical Services
GCP: Good clinical practice
IEPs: Incentives and Earned Privileges
IME: Important medical event
IMP: Investigational medicinal product
KHPCTO: King’s Health Partners Clinical Trials Office
MAR: Missing at random
MEWS: Mind Excessively Wandering Scale
MHRA: Medicines and Healthcare products Regulatory Agency
MINI: International Neuropsychiatric Interview for common mental health disorders
MOASE: Modified Overt Aggression Scale by Education Staff
MOASP: Modified Overt Aggression Scale by Prison Officers
MPH: Methylphenidate
MRC: Medical Research Council
MVQ: Maudsley Violence Questionnaire
NICE: National Institute for Health and Care Excellence
NIDA: National Institute on Drug Abuse
REC: Research ethics committee
RPAQ: Reactive-Proactive Aggression Questionnair
SAE: serious adverse events
SAR: serious adverse reactions
SIGN: Scottish Intercollegiate Guidelines Network
SMD: Standardised mean difference
SUSAR: suspected unexpected serious adverse reactions
TSC: Trial steering committee
WASI: Wechsler Abbreviated Scale of Intelligence
Weiss-CD: Weiss Conduct Disorder Scale
WRAADS: Wender-Reimherr Adult ADHD Diagnostic Scale
ZAB-BPD: Zanarini Rating Scale for Borderline Personality Disorder
