# Supplementary material for: Randomised controlled trial of the short-term effects of OROS-methylphenidate on ADHD symptoms and behavioural outcomes in young male prisoners with attention-deficit/hyperactivity disorder (CIAO-II)
Source: Trials. 2019 Dec 2;20:663. doi: 10.1186/s13063-019-3705-9 (PMC6889577; doi:10.1186/s13063-019-3705-9)
Supplement: Supplementary file 1 — Additional file 1. : Information sheet and consent form for the screening stage and trial stage of the study. (DOCX 44 kb) [file 13063_2019_3705_MOESM1_ESM.docx]

**Participant Information Sheet**

**The First stage – CIAO-II (ADHD) Project**

**Title of Project:** Randomised controlled trial of the short-term effects of OROS-methylphenidate on ADHD symptoms and behavioural outcomes in young male prisoners with attention deficit hyperactivity disorder

You are invited to take part in a research study of attention deficit hyperactivity disorder (ADHD). If you agree to take part there are two main stages:

- We will ask you some questions about the way you are. The questions will ask about problems with paying attention, feeling fidgety or restless and doing things quickly, without thinking ahead.
- After the assessment if it turns out that you have ADHD you will be invited to take part in a clinical trial of an ADHD medication called Concerta XL. You will be given more information about this if it turns out that you have ADHD.

**This information sheet is about the first stage of the study.**

Please take time to read this information about the study and ask any questions. Take your time to decide whether you wish to take part in the study. Taking part in this study is your choice - it will not affect your prison sentence or health care if you decide you do not wish to take part. Thank you for reading this.

**What is the study about?**

You are invited to take part in a research study looking at the treatment of ADHD in prison. We know from earlier research that around 1 in 5 people in prison have ADHD without being offered treatment.

If we find that you have ADHD we will offer you treatment. You will be invited to take part in a clinical trial of medication for ADHD or you can be helped by the prison health care team.

**What is ADHD?**

ADHD is a problem that usually starts when you are a young child and causes problems with concentration and being too active or impulsive. Lots of children grow out of ADHD but 1 in 50 adults in England still have ADHD and more people in prisons.

People with ADHD often feel irritable, frustrated, aggressive or angry, as well as having problems with their concentration, being forgetful or losing things and finding it difficult to plan and organise their day. In prison people with ADHD may find it difficult to manage work activities, get bored and frustrated very quickly or have times when they can’t control their anger.

**Why me?**

You have been invited because you are a prison inmate here. We are inviting all prison inmates to answer some questions about ADHD. If the answers suggest that you may have ADHD, you will be asked if you want to have a further assessment.

**Do I have to do it?**

No, it is up to you to decide whether you want to do it or not. Taking part in this study or not will have no effect on your care in the prison or the length of your prison sentence. Even if you say yes first, you can still stop doing it later on, if you want to.

**What would I do if I took part?**

If you decide to take part, we will ask you to sign a consent form. You will be asked some questions which takes around 5 minutes. The research team will also look at your prison and health care records.

**What would I get out of it?**

If we find out that you have ADHD you will be invited to take part in a clinical trial. This may benefit you since you will receive study medication and meet with the research team every week. If you take part in the clinical trial you will be given either Concerta XL or a dummy pill called a placebo each day for 8 weeks. After that we will be able to continue to treat you for ADHD.

If you do not wish to take part in the clinical trial we can refer you to the prison mental health team for treatment of ADHD.

**What are the downsides to taking part?**

It will take up some of your time. We will ask you questions about yourself and some people might find this difficult or get upset.

**Confidentiality**

If you agree to take part in this project, the research team will look at your medical and prison records. This is because we wish to find out more about the way ADHD affects people in prison and the way they manage in education and work.

All personal information about you will be strictly confidential and will be stored in a secure place. You will be given a study ID code and only the research team will be able to match your name and prison number with your answers to the questions.

King’s College London is the sponsor for this study based in the United Kingdom. We will be using information from you and your medical records in order to undertake this study and will act as the data controller for this study. This means that we are responsible for looking after your information and using it properly. King’s College London will keep identifiable information about you for 5 years after the study has finished. Your right to access, change or move your information are limited, as we need to manage your information in specific ways in order for the research to be reliable and accurate. If you withdraw from the study, we will keep the information about you that we have already obtained. To safeguard your rights, we will use the minimum personally-identifiable information possible. You can find out more about how we use your information on the link (<https://www.kcl.ac.uk/governancezone/assets/governancelegal/data-protection-policy.pdf> ).

The only times the research team would need to break this confidentiality and disclose information to prison staff is if you were to make a threat by intending to harm yourself or someone else, were a threat to security, behaved in a way that is against the prison rules and can be adjudicated against, or any other illegal acts. We will also have to report any information that relates to a previous crime that was not reported before, and information about any drugs you may be taking that were not prescribed by the health care team.

**What will happen afterwards?**

The information collected will be analysed by Professor Philip Asherson and the research team at King’s College London, Professor Lindsay Thomson at the University of Edinburgh.

The results of the study will be published in science journals. All personal information will remain strictly confidential so you will not be recognised in any of the research reports. If you want we can send you a report of the study findings after the research has been completed.

Research data in a form that cannot be traced back to you as an individual may be shared with other scientists or research groups who will assist in analysing the data, in order to better understand treatment of ADHD in prisoners. At the end of the study all research records will be transferred to King’s College London using an approved courier for a secure service. Consent forms will be separate from all other records to ensure that the data is not identifiable.

**Who is doing this research?**

This research is organised by the Professor Asherson and his team at the Institute of Psychiatry, Psychology and Neuroscience, King’s College London and [trust name].

**Who is paying for this research?**

The research is funded by the National Institute for Health Research, the main NHS funder in the UK. Janssen-Cilag who makes Concerta XL provided the medication for the study.

**Who has approved this study?**

The study has Ethical approval by the NRES Committee [xxxxxx]. It is a clinical trial and is guided by the regulations for clinical trials in the UK.

**What do I do now?**

We hope that you will be able to help in this important project. Please feel free to ask any questions. If you want to take part in the study you need to sign the consent form.

**Independent advice:**

Patient Liaison Service telephone: [Local trust email, telephone number, address].

**What if there is a problem?**

If you have any concerns you should speak to someone in the research team who will do their best to help. The research team includes a qualified mental health specialist who can provide advice if you experience any distress, anxiety, depression or other health problem while taking part in the study.

If you remain unhappy and wish to complain formally, you can do this by contacting the Trust's Complaints Department: [Local trust email and telephone number, post address]. If something goes wrong and you are harmed during the research and this is due to someone‘s carelessness you could take legal action for compensation against the Trust. However, you may have to pay your legal costs. You may also make complaints using the prisoner complaints procedures.

**Contact details:**

If you want more information about the project please contact a member of the research team who are based in prison healthcare.

If you wish to make a complaint you can contact the research team, the prison healthcare team or follow the usual prison procedures for prisoner complaints.

**CONSENT FORM- CIAO-II (ADHD) Project**

Title of Project: Randomised controlled trial of the short term effects of OROS-methylphenidate on ADHD symptoms and behavioural outcomes in young male prisoners with attention deficit hyperactivity disorder

**Participant ID: Study Number: Please initial box**

1. I have read and understand the information sheet for this project and have had the chance to ask questions. The information sheet is dated 28th of August 2018, version 2.0.
2. I understand that taking part is my choice. I am free to stop at any time. I don’t have to give a reason. My medical care or legal rights won’t be affected.
3. I understand that parts of my medical notes may be looked at. This is to confirm diagnosis of ADHD and check I can take part in the project. I agree for Professor Asherson and his research team to have access to my medical and prison records. To enable monitoring of the conduct of the study, I give permission for regulatory authorities, sponsor representative or individuals from the [trust name] to access my clinical and research records.
4. I agree to take part in this project.
5. I agree that my information from this study will be stored securely. It can be shared with other scientists or research groups where this helps us to understand the findings of the study. My name and other identifying information will be kept separate. The information can be used in combination with data from other similar studies. In the event of publication, I understand that care will be taken to keep my personal information private. I understand that the research records will be transferred securely to King’s College London at the end of the study.
6. I agree for my personal contact details to be kept securely by the research team. I give permission to be contacted in the future about further research projects related to the treatment of ADHD. I can remove my name and details from this list at any time and without giving a reason.

________________________ ____________________ ____________________

Name of Participant Date Signature

_________________________ ____________________ ____________________

Name of Person taking consent Date Signature

(If different from Researcher)

I have explained the study to the participant and have answered their questions honestly and fully.

_________________________ _____________________ ____________________

Researcher Date Signature

**The Second stage – CIAO-II (ADHD) Project**

**Title of Project:** Randomised controlled trial of the short term effects of OROS-methylphenidate on ADHD symptoms and behavioural outcomes in young male prisoners with attention deficit hyperactivity disorder

You are invited to take part in a research study of attention deficit hyperactivity disorder (ADHD). You have already taken part in the first stage of the study in which we were able to confirm the diagnosis of ADHD. We are now inviting you to take part in the treatment stage – a clinical trial of an ADHD medication called Concerta XL.

Please take time to read this information about the study and ask any questions. Take your time to decide whether you wish to take part in the study. Taking part in this study is your choice - it will not affect your prison sentence or health care if you decide you do not wish to take part. Thank you for reading this.

**What is ADHD?**

ADHD is a problem that usually starts in early childhood and causes problems with concentration and being too active or impulsive in everyday situations. For example being too angry, talking too much, interrupting people all the time or feeling irritable when having to wait in queues. People with ADHD are often disorganised in the way they go about things and find it difficult to start or complete tasks, complete paper forms, manage their money and focus on jobs or education activities.

ADHD affects about 1 in 50 adults in the United Kingdom, and as many as 1 in 5 people in prisons.

**What is this study about?**

We want to find out whether a medication for ADHD called methylphenidate, or MPH for short, can help people with ADHD in prison.

MPH is the usual treatment for ADHD. Most people have heard of Ritalin which is sometimes used to treat people with ADHD. Ritalin is in fact the same as MPH. There are other drugs also made of MPH, including one called Concerta XL which we will use in this study.

**What is the study medication?**

Concerta XL is one of the most common drugs used to treat ADHD in the United Kingdom. Concerta XL is not licensed to be used in adults that have never used it as children. However Concerta XL is recommended for the treatment of ADHD by the National Institute for Clinical Excellence (NICE) in England. In Scotland, there is no specific recommendation for the first time treatment of ADHD in adults over the age of 17, but the NICE guidelines are widely accepted across the UK. This means that doctors have been told that this drug can be used to treat adults with ADHD, even though this is outside of its licensed use.

**Why me?**

We are inviting you to take part in this study because your assessment in the first stage of this study found that you have ADHD. We are inviting all people who have ADHD and who might benefit from treatment to take part in this study.

**Do I have to do it?**

No, it is up to you to decide whether you want to do it or not. Taking part in this study or not will have no effect on your care in the prison or the length of your prison sentence. Even if you say yes first, you can still stop doing it later on, if you want to. If you do not wish to take part in the clinical trial we can refer you to the prison mental health team for treatment of ADHD

It is potentially dangerous to mix the study medication with other drugs. It is therefore important that you do not take part in the study if you are taking any drugs or medication other than those provided by the health care team. If we discover that you are taking drugs that were not prescribed by the health team we are obliged to report this.

**What would I do if I took part?**

If you take part in this study you will be given either Concerta XL or a dummy pill called a placebo each day for 8 weeks. We will check to make sure that any other medication you take does not interact with Concerta XL in a bad way.

You or anyone in the medical team will **not** know whether you will have the drug or the dummy pill. A computer at the Kings College London Clinical Trials Unit will decide what you will be getting and the pills will look and taste the same. You have a one in two chance of getting Concerta XL or the dummy pill – so the chance of getting the drug or the dummy pill is like tossing a coin.

You will be given the study medication each day. The effects of Concerta XL last around 10 hours and then wear off. The most common effect is to be able to concentrate better, be more alert, feel calmer and in control of your thoughts and feelings. It should not make you feel sleepy. You will have weekly medical assessments for the first 5 weeks, and then three weeks later at the end of the study. We will check to see if there are any side effects that you do not like or might be dangerous to your health. We will check your pulse and blood pressure and ask you about problems related to ADHD.

During the first 5 weeks we will slowly increase the dose of the study medication to find the dose that is best for you. After the first 5 weeks the dose will then remain the same.

We will ask you questions about your ADHD and ask you to complete some forms about how you are feeling and managing in the prison (we can help you with this). This will be done at the following times:

- before starting the medication
- once a week for the first 5 weeks of the study
- after eight weeks of the study

At the end of the trial you will be asked whether you wish to continue to be treated for ADHD. Whether you were taking Concerta XL or the dummy medication, we will be able to treat you with medication for ADHD. We will also help you to obtain your medication once you leave the prison.

**How much time will this take?**

There is a lot to do if you take part in this study. We will see you seven times or more during the study. The first time we see you it will take about 2 hours. Once you start the study medication we will see you for the next 5 weeks for about 30-minutes.

At the end of the study, after 8 weeks, we will see you around 1 ½ hours and ask you some of the same questions we asked at the beginning of the study, so we can see how the medication may have helped you.

We think it will take a total of 6 hours of your time over an 8-weeks period to be in this study to the end.

**What are the possible benefits of taking part?**

By taking part in this study we will find out whether Concerta XL can help people in prison with problems related to ADHD.

Some of the benefits include:

- Improved concentration
- Improved ability to focus on tasks and not get distracted
- Having a less busy and more focused mind
- Feeling less restless and fidgety
- Being more patient when waiting
- Being less irritable or angry
- Finding it easier to work with other people and engage in work activities
- Feeling more calm and better able to control emotions such as feeling too irritable or angry or having mood swings
- Some people find it easier to get off to sleep.

Once the 8-week trial is over we will continue to treat you for ADHD during your stay in the prison.

**What are the possible disadvantages and risks of taking part?**

Like all drug treatments, Concerta XL can cause side effects.

Many of the common side effects stop after 1 or 2 weeks, however some may continue. You may also have side effects that stop you using Concerta XL because they are not nice to experience or have a bad effect on your health. In some rare cases more severe side effects can occur that would lead us to stop the drug immediately.

**Side effects go from very common to very rare. They include the following:**

- **Very common side effects:** Affect more than 1 in 10 people. Include headache, nervous feelings and difficulty sleeping.
- **Common side effects**: Less than 1 in 10 people. Include dizziness, drowsiness, blurred vision, loss of appetite, weight loss, increased aggression or hostility, stomach pain or upset, inability to develop or maintain an erection, heart palpitations, increased blood pressure and heart rate.
- **Uncommon side effects:** Less than 1 in 100 people. Concerta XL can cause chest pain, hallucinations or delusions, worsening of tics, blood disorders or inflammation of blood vessels in the brain.
- **Rare side effects**: Less than 1 in 1,000 people may feel unusually excited, out of control or manic.
- **Very rare side effects**: Less than 1 in 10,000 people have very serious side effects. Those that have been reported include heart attack, sudden death and liver failure.

You will be monitored closely by the health staff to ensure that any side effects are found early and treated quickly. If serious side effects occur, the medication will be discontinued immediately. If at any time you think you may have side effects or unwanted effects of the drug, you should contact the prison health team.

During the assessment we will ask you about problems related to ADHD and other health problems. Some of the questions we ask you about yourself may bring up sensitive issues. If you become upset and wish to talk to someone, we will tell you where you can get support and advice.

**Confidentiality**

If you agree to take part in this project, the research team will look at your medical and prison records. We will also be talking to prison and education staff to find out how you have been doing. This is because part of the research is to see whether treatment for ADHD can help you with your education and work programme, and to see whether you experience fewer problems like becoming irritable, angry or aggressive (if you have this problem).

All personal information about you will be strictly confidential and will be stored in a secure place. You will be given a study ID code and only the research team will be able to match your name and prison number with your answers to the questions.

King’s College London is the sponsor for this study based in the United Kingdom. We will be using information from you and your medical records in order to undertake this study and will act as the data controller for this study. This means that we are responsible for looking after your information and using it properly. King’s College London will keep identifiable information about you for 5 years after the study has finished. Your right to access, change or move your information are limited, as we need to manage your information in specific ways in order for the research to be reliable and accurate. If you withdraw from the study, we will keep the information about you that we have already obtained. To safeguard your rights, we will use the minimum personally-identifiable information possible. You can find out more about how we use your information on the link (<https://www.kcl.ac.uk/governancezone/assets/governancelegal/data-protection-policy.pdf> ).

Other authorised people may need to look at your medical and prison records to check that the study is being carried out correctly.

The only times the research team would need to break this confidentiality and disclose information to prison staff is if you were to make a threat by intending to harm yourself or someone else, were a threat to security, behaved in a way that is against the prison rules and can be adjudicated against, or any other illegal acts. We will also have to report any information that relates to a previous crime that was not reported before, and information about any drugs you may be taking that were not prescribed by the health care team.

**What will happen to the results of the research study?**

The information collected will be analysed by Professor Philip Asherson and the research team at King’s College London, Professor Lindsay Thomson at the University of Edinburgh.

The results of the study will be published in science journals. All personal information will remain strictly confidential so you will not be recognised in any of the research reports. If you want we can send you a report of the study findings after the research has been completed.

Research data in a form that cannot be traced back to you as an individual may be shared with other scientists or research groups who will assist in analysing the data, in order to better understand treatment of ADHD in prisoners. At the end of the study all research records will be transferred to King’s College London using an approved courier for a secure service. Consent forms will be separate from all other records to ensure that the data is not identifiable.

**Who is organising the research?**

This research is organised by Professor Asherson and his team at the Institute of Psychiatry Psychology and Neuroscience, King’s College London and [trust name].

**Who is paying for this research?**

The research is funded by the National Institute for Health Research, the main NHS funder in the UK. Janssen-Cilag who makes Concerta XL provided the medication for the study. The study is being led by Professor Asherson and his team at the Institute of Psychiatry, Psychology and Neuroscience, King’s College London.

**Who has approved this study?**

The study has Ethical approval by the NRES Committee [xxxxxx] It is a clinical trial and is guided by the regulations for clinical trials in the UK.

**What do I do now?**

We hope that you will be able to help in this important project. Please feel free to ask any questions. If you want to take part in this study you need to sign the consent form.

You may ask to see one of the research team by contacting the prison healthcare team. You may also call the telephone number [XXX] to speak to the study coordinator.

**Independent advice:**

Patient Liaison Service telephone: [Local trust email, telephone number, address]

**What if there is a problem?**

If you have any concerns you should speak to someone in the research team who will do their best to help. The research team includes a qualified mental health specialist who can provide advice if you experience any distress, anxiety, depression or other health problem while taking part in the study.

If you remain unhappy and wish to complain formally, you can do this by contacting the Trust's Complaints Department: [Local trust email and telephone number, post address].If something goes wrong and you are harmed during the research and this is due to someone‘s carelessness you could take legal action for compensation against the Trust. However, you may have to pay your legal costs. You may also make complaints using the prisoner complaints procedures.

**Contact details:**

If you want more information about the project please contact a member of the research team who are based in prison healthcare.

If you wish to make a complaint you can contact the research team, the prison healthcare team or follow the usual prison procedures for prisoner complaints.

**CONSENT FORM- CIAO-II (ADHD) Project**

**Title of Project:** Randomised controlled trial of the short term effects of OROS-methylphenidate on ADHD symptoms and behavioural outcomes in young male prisoners with attention deficit hyperactivity disorder

**Participant ID: Study Number: Please initial box**

1. I have read and understand the information sheet for this project and have had the chance to ask questions. The information sheet is dated 28^th^ of August 2018, version 2.0.
2. I understand that taking part is my choice. I am free to stop at any time. I don’t have to give a reason. My medical care or legal rights won’t be affected.
3. I understand that parts of my medical notes may be looked at. This is to confirm diagnosis of ADHD and check I can take part in the project. I agree for Professor Asherson and his research team to have access to my medical and prison records. To enable monitoring of the conduct of the study, I give permission for regulatory authorities, sponsor representative or individuals from the [trust name] to access my clinical and research records.
4. I agree to take part in this project.
5. I agree that my information from this study will be stored securely. It can be shared with other scientists or research groups where this helps us to understand the findings of the study. My name and other identifying information will be kept separate. The information can be used in combination with data from other similar studies. In the event of publication, I understand that care will be taken to keep my personal information private. I understand that the research records will be transferred securely to King’s College London at the end of the study.
6. I agree for my personal contact details to be kept securely by the research team. I give permission to be contacted in the future about further research projects related to the treatment of ADHD. I can remove my name and details from this list at any time and without giving a reason.

________________________ ____________________ ____________________

Name of Participant Date Signature

_________________________ ____________________ ____________________

Name of Person taking consent Date Signature

(If different from Researcher)

I have explained the study to the participant and have answered their questions honestly and fully.

_________________________ _____________________ ____________________

Researcher Date Signature
